# Supplementary material for: HIVprotI: an integrated web based platform for prediction and design of HIV proteins inhibitors
Source: J Cheminform. 2018 Mar 9;10:12. doi: 10.1186/s13321-018-0266-y (PMC5845081; doi:10.1186/s13321-018-0266-y)
Supplement: Supplementary file 2 — Additional file 2. Source code of HIVProtI web server. [file 13321_2018_266_MOESM2_ESM.zip › HIVprotI_Source-code/hivprotI/weka-3-6-6/doc/weka/filters/supervised/attribute/package-summary.html]

weka.filters.supervised.attribute


---


|  |  |  |
| --- | --- | --- |
| **Overview** \| **Package** \| Class \| **Tree** \| **Deprecated** \| **Index** \| **Help** \| **Weka's home** | |  |
| **PREV PACKAGE**   **NEXT PACKAGE** | **FRAMES**    **NO FRAMES**     **All Classes** |  |


---

## Package weka.filters.supervised.attribute

| **Class Summary** | |
| --- | --- |
| **AddClassification** | A filter for adding the classification, the class distribution and an error flag to a dataset with a classifier. |
| **AttributeSelection** | A supervised attribute filter that can be used to select attributes. |
| **ClassOrder** | Changes the order of the classes so that the class values are no longer of in the order specified in the header. |
| **Discretize** | An instance filter that discretizes a range of numeric attributes in the dataset into nominal attributes. |
| **NominalToBinary** | Converts all nominal attributes into binary numeric attributes. |
| **PLSFilter** | Runs Partial Least Square Regression over the given instances and computes the resulting beta matrix for prediction.  By default it replaces missing values and centers the data.    For more information see:    Tormod Naes, Tomas Isaksson, Tom Fearn, Tony Davies (2002). |

---


|  |  |  |
| --- | --- | --- |
| **Overview** \| **Package** \| Class \| **Tree** \| **Deprecated** \| **Index** \| **Help** \| **Weka's home** | |  |
| **PREV PACKAGE**   **NEXT PACKAGE** | **FRAMES**    **NO FRAMES**     **All Classes** |  |


---
